# The Consumption of Dairy Products Is Associated with Reduced Risks of Obesity and Metabolic Syndrome in Korean Women but not in Men

**DOI:** 10.3390/nu9060630

**Published:** 2017-06-19

**Authors:** Kyung Won Lee, Wookyoun Cho

**Affiliations:** 1 Department of Food Science and Human Nutrition, Michigan State University, 469 Wilson Road, Trout FSHN Building, East Lansing, MI 48824, USA; kyungwon@msu.edu; 2 Department of Food and Nutrition, Gachon University, 1342 Seongnam-daero, Sujeong-gu, Seongnam-si, Gyeonggi 13120, Korea

**Keywords:** dairy products, milk, obesity, metabolic syndrome, KNHANES

## Abstract

We aimed to investigate the association between dairy product consumption and the risk of obesity and metabolic syndrome (MetS) in Korean adults. Data from 13,692 Korean adults aged ≥19 years from the KNHANES 2010–2013 were used. The study participants were divided into three groups according to the serving size of dairy products they consumed based on a single 24-h recall. About 58% of the Korean adults did not consume any dairy products in one day. In both the sexes, only those who adhered to the recommendation for dairy products (≥1 serving/day) achieved the daily requirement of calcium. Women who consumed ≥1 serving/day of dairy products had lower risks of obesity (body mass index ≥ 25 kg/m^2^) (adjusted odds ratio (AOR), 0.77; 95% confidence interval (CI), 0.66–0.89; *p* for trend < 0.01) and MetS (AOR, 0.67; 95% CI, 0.56–0.80; *p* for trend < 0.01) than women who did not consume dairy products. However, these significant associations were not observed in men. In conclusion, consuming ≥ 1 serving/day of dairy products could be an easy and efficient strategy for meeting daily calcium requirement as well as lowering risks of obesity and MetS among Korean women.

## 1. Introduction

Dairy products have long been considered a superfood, as they are a source of calcium, high-quality protein, vitamin B_2_, vitamin D, potassium, and medium-chain fatty acids [1]. Accordingly, the intake of dairy products has been widely recommended, and many countries recommend the consumption of dairy products as a component of a healthy diet [2,3]. However, there have been concerns about excessive saturated fatty acid and caloric intake from dairy products; thus, the 2010 Dietary Guidelines for Americans currently recommends 2–3 cups of milk per day in nutrient-dense forms, including fat-free/low-fat milk and yogurt, to prevent cardiovascular disease [4].

According to 2011 statistics by the Food and Agriculture Organization of the United Nations, the global average milk supply was 90.3 kg/capita, while it was 253.0 kg/capita in the United States (US) alone [5]. The consumption of milk, yogurt, and cheese has held an important position in the Western diet, which includes a particularly high consumption of dairy products. On the other hand, the average milk supply was 57.8 kg/capita for Asian countries, which have a traditionally low consumption of dairy products in diets; for Korea, this average is even lower, at 26.4 kg/capita. A recent report showed that the amount of milk consumed in Korea has increased from 79.7 g in 1999 to 120.7 g in 2013. However, only 20% of adults aged 19–64 years consume milk more than once a day, and an inverse correlation was noted recently between decreased milk consumption and the increasing age of both men and women [6]. The lower consumption of dairy products has, for a long time, been closely linked, in the literature, to an inadequate intake of dietary calcium and vitamin D among Koreans [7,8].

Previous works have noted that consuming one serving of milk and dairy products a day was the most efficient way for Koreans to consume 200 mg of calcium, which corresponds to nearly one third of the daily calcium requirement [9]. Based on this favorable role of dairy products in dietary calcium intake, the 2010 Dietary Reference Intakes (DRI) for Koreans recommends two cups of milk for adolescents and one cup of milk for adults per day. However, the consumption of dairy products among Koreans has failed to meet this recommendation [9,10].

Numerous cross-sectional and prospective studies from the US and Europe have suggested that the consumption of dairy products was protective against the development of obesity and metabolic syndrome (MetS) by affecting more than one of the risk factors of these diseases, even though some studies have reported no association, as well as disparate effects of dairy product consumption between men and women [11,12]. In addition to the studies conducted in Western countries, similar studies have been conducted in Asian countries, where the consumption of dairy products has occupied a less important position, relatively. A study of Japanese adults aged 20–68 years reported that more than one serving of full-fat dairy, but not low-fat dairy, per day decreased the risk of insulin resistance [13]. Additionally, a large-scale cohort study of Japanese adults observed an inverse association between dairy product consumption and type 2 diabetes mellitus, but only in women [14]. A recent study conducted in China showed that dairy product consumption was associated with a lower risk of type 2 diabetes mellitus and favorable changes of waist circumference (WC), body mass index (BMI), and blood pressure [15].

Several population-based studies among Koreans have also found beneficial effects of dairy product consumption, measured by food-frequency questionnaires (FFQs), on components of MetS [16,17]. Considering that the prevalence of MetS is increasing dramatically in Korean adults [18], understanding the beneficial metabolic effects of dairy product consumption is essential in future policy design. However, the FFQs used in those studies have not been adequately validated [19], i.e., they only measured the frequency of dairy product consumption, but not the amount and serving size of each dairy product consumed. Thus, it might be too early to conclude that dairy product consumption is negatively associated with MetS among Koreans, based on studies using insufficiently validated FFQs. To fill this gap, it is necessary to conduct a systematized and validated study using different collecting methods that may accurately reflect the amount of dairy product consumed. Additionally, previous Korean studies reported differences between men and women in the prevalence of MetS and the combinations of its individual components [18,20]. However, there is limited evidence regarding the differences between the sexes in the effects of dairy product consumption with regards to the many components of MetS.

Therefore, this study attempted to test whether consuming more than the daily-recommended amount of dairy products may reduce the risk of obesity and MetS in the Korean adult population using nationally representative data. Accordingly, the aims of this study were to describe the dairy product consumption status, and to investigate the association of the consumption of dairy products with nutrients and food intake, and the risk of obesity and components of MetS between the sexes in Korean adults.

## 2. Methods

### 2.1. Data Source and Study Participants

The Korea National Health and Nutrition Examination Survey (KNHANES) is a continuous nation-wide system of surveillance conducted by the Korea Centers for Disease Control and Prevention (KCDC) and the Korean Ministry of Health and Welfare. The KNHANES uses a multi-stage clustered sampling design to collect representative data on the health and nutritional status of non-institutionalized Korean citizens. The survey also allows investigators to explore associations between nutritional intake, health-related behaviors, and health outcomes. The protocols and procedures of the KNHANES were reviewed and approved by the KCDC Institutional Review Board (2010-02CON-21-C, 2011-02CON-06-C, 2012-01EXP-01-2C, 2013-07CON-03-4C), and all participants gave their written informed consents [21,22].

The study participants consisted of Korean adults aged 19–64 years with nutrition survey (*n* = 18,888) who participated in the KNHANES 2010–2013. We excluded those with implausible total energy intakes (<500 or >5000 kcal/day [23]; *n* = 2718) and pregnant/lactating women (*n* = 249). We also excluded individuals with incomplete information on socio-demographic characteristics (*n* = 1528), health-related behaviors (*n* = 88), and biomarkers (*n* = 613) for a final analytic sample of 13,692 (5375 men and 8317 women).

### 2.2. Dietary Assessment

Dairy product consumption was determined from 24-h dietary recalls based on the KNHANES coding scheme [21] and previous literature [24,25] for whole-fat milk, reduced fat (2%)/low-fat (1%) milk, sweetened milk, yogurt (which includes both liquid and semi-solid types), cheese/cheese products, and ice cream/dairy-based desserts. Based on the distributions of dairy product consumption of the study participants, the following categories were created: non-consumers (0 servings/day), 0< to <1 serving/day, and ≥1 serving/day (which is equivalent to the daily recommendation of dairy product consumption for Korean adults) [9].

The 24-h dietary recall data on all foods and beverages consumed were also used in this study to estimate energy and nutrient intakes and food consumption across the dairy product consumption levels. We calculated all the study participants’ total daily energy intake (kcal), the intake amount (g) of and % energy from macronutrients (carbohydrate, protein, and fat) and the amount of dietary fiber (g), sodium (mg), potassium (mg), phosphorus (mg), and calcium (mg). In the case of calcium, we estimated calcium intake from dairy products and other sources, respectively, as well as total calcium intake from all dietary sources. Since the amount of each food group consumed in the 24-h dietary recalls allows us to understand food consumption patterns [26], we also investigated food group intakes according to dairy product consumption. The food groups were categorized based on the food code system used in the KNHANES food database [21] and prior studies [24,27]. All food consumed by study participants from the KNHANES were grouped as whole grains, refined grains, noodles/dumplings, flour/breads, burgers/pizza, starch vegetables, other vegetables, fruits, meat/poultry, fish/shellfish, eggs, legumes/legume products, nuts/seeds, sugars/sweets, oils/fats, dairy products, non-alcoholic beverages, alcoholic beverages, and others.

### 2.3. Obesity and MetS

The weight, height, and waist circumference of the study participants were directly measured to the nearest 0.1 kg and 0.1 cm. Blood samples were obtained after more than 8 h of fasting according to standard protocols [21]. The BMI was calculated as weight (kg)/height (m^2^). Obesity was defined as BMI ≥ 25 kg/m^2^ based on the criteria for the Asia-Pacific region of the World Health Organization [28]. MetS was defined using the criteria of National Cholesterol Education Program Adult Treatment Panel III (NCEP ATP III) [29] and the International Diabetes Federation [30], which diagnoses MetS on the basis of at least three of the following risk factors: (1) abdominal obesity (WC ≥ 90 cm in men and ≥80 cm in women); (2) elevated triglyceride (TG) (fasting TG ≥ 150 mg/dL or specific treatment for hypertriglyceridemia); (3) low HDL cholesterol (HDL-C) (fasting HDL-C < 40 mg/dL in men and <50 mg/dL in women); (4) elevated fasting blood glucose (FBG) (FBG ≥ 100 mg/dL or specific treatment for/previously diagnosed with type 2 diabetes mellitus); and (5) elevated blood pressure (systolic blood pressure ≥ 130 mmHg or diastolic blood pressure ≥ 85 mmHg or specific treatment for/previously diagnosed with hypertension).

### 2.4. Potential Covariates

The following data regarding sociodemographic characteristics and health-related behaviors were collected for Korean adults over 19 years of age: sex (men and women); age (19–29, 30–49, and 50–64 years); income (lowest, lowest middle, upper middle, and highest; income was classified into quartiles of the equalized household income which was calculated from the total household income divided by the square root of the number of household members); education level (middle school graduates or less, high school graduates, and college graduation or higher); smoking status (non-/former smoker or current smoker), alcohol consumption (never/rarely, 1–4 times/month, and ≥2 times/week; alcohol consumption was classified based on the question, “How often during the last year have you had alcoholic drinks?”), and physical activity (yes or no; physical activity was defined as walking ≥ 5 time a week for ≥30 min each time).

### 2.5. Statistical Analysis

To account for the multi-stage stratified probability sampling of the KNHANES, the PROC SURVEY with the sample weights, stratum, and primary sampling unit, as provided by the KCDC [21], were used in all data analyses using SAS (version 9.4, SAS Institute Inc., Cary, NC, USA). All statistical tests were two-tailed and statistical significance was set at *p* < 0.05.

Sociodemographic characteristics, health-related behaviors, and the prevalence of obesity, and MetS were compared across the levels of dairy product consumption using the chi-square test. Multiple linear regressions were performed to investigate the association between dairy product consumption with intakes of energy, nutrients, and food groups. These regression models were controlled for covariates including age (continuous), income, education level, smoking status, alcohol consumption, and physical activity. For dietary fiber, sodium, potassium, phosphorus, and calcium, we additionally adjusted for total energy intake (continuous). Logistic regressions were conducted to determine the associations between dairy product consumption with odds of obesity and MetS. First, we confirmed the unadjusted odds ratios (ORs) with 95% confidence intervals (CIs) using logistic linear regressions (crude model). We then calculated the multivariable-adjusted ORs with 95% CIs after controlling for covariates in two models: model 1 was adjusted for age (continuous), income, education level, smoking status, alcohol consumption, and physical activity, and model 2 was adjusted for the same covariates as model 1 along with the inclusion of total energy intake (continuous). Non-consumers of dairy products were considered a reference in all the regression models.

## 3. Results

About 60% of the Korean adult population (*n* = 8079) did not consume dairy products, with only about 26% (*n* = 3534) consuming dairy products at or above the recommended level (≥1 serving) per day (Table 1). Those consuming more than the recommended level per day were more likely to be women, highly educated, and non-smokers and less likely to be older than those who consumed <1 serving per day (all, *p* < 0.01). Those who never or rarely drink alcohol and undertake regular physical activity also had higher consumption of dairy products (*p* < 0.01).

Energy and nutrient intake by dairy product consumption are shown in Table 2. A higher consumption of dairy products was associated with energy intake in men (*p* < 0.01) and women (*p* < 0.01). In both sexes, dairy product consumption was positively related with the intake amount of macronutrients (carbohydrates, protein, and fat), dietary potassium, and phosphorous but negatively related with the percentage of energy from carbohydrates and dietary sodium (all, *p* < 0.01). No associations between dietary fiber intake and the consumption of dairy products were observed in men and women. Interestingly, there were dramatic differences in the intakes of dietary calcium via dairy product consumption in both sexes. Higher dairy product consumption was correlated with increased total dietary calcium and calcium intake from dairy products, but with a decrease in dietary calcium from non-dairy sources (all, *p* < 0.01). Food group intakes according to dairy product consumption are shown in Table 3. Higher consumption of dairy products was associated with a greater intake of flour and breads, and a lower intake of refined grains, meat and poultry, alcoholic beverages, oils/fats, vegetables, and fish and shellfish (all, *p* < 0.01). Differences in the food group consumption between men and women were found only in non-alcoholic beverages and sugars/sweets. The increment in the consumption of those food groups across dairy product consumption was significantly different in men (*p* < 0.05); however, in women, there was no difference among dairy product consumption groups.

The prevalence of obesity and the components of MetS by sex and dairy product consumption are listed in Table 4. Among both men and women, the highest prevalence of MetS was found in non-consumers of dairy products in both sexes (men: 25.23%, *p* < 0.01; women: 23.76%, *p* < 0.01). The multivariable-AORs for obesity and risk factors of MetS according to dairy product consumption by sex are shown in Table 5. Women consuming ≥ 1 serving/day of dairy products were less likely to be at risks of obesity (AOR: 0.77; 95% CI: 0.66–0.89; *p* for trend < 0.01), abdominal obesity (AOR: 0.71; 95% CI: 0.62–0.82; *p* for trend < 0.01), lowered HDL-C (AOR: 0.82; 95% CI: 0.72–0.93; *p* for trend < 0.01), elevated FBG (AOR: 0.80; 95% CI: 0.67–0.95; *p* for trend < 0.01), and MetS (AOR: 0.67; 95% CI: 0.56–0.80; *p* for trend < 0.01) than women who did not consume any dairy products. However, there was no significant association between greater dairy product consumption and the risk of obesity and MetS components in men.

## 4. Discussion

Our findings showed that only 25.8% of Korean adults (23.3% men and 27.4% women) consumed dairy products at the recommended level (1 serving/day). We also found that those consuming dairy products above the recommended level tended to be non-smokers or former smokers, never or rarely consumed alcohol, and participated in regular physical activities, all of which are usually associated with healthier lifestyles. Our findings were congruent with the previous study indicating that dairy product consumption might be an indicator of healthy behavior or a healthy option for disease prevention [32,33].

Interestingly, the most striking difference in nutrient intakes according to the consumption of dairy products was shown in dietary calcium. According to the DRI for Koreans, people of both sexes who consumed <1 serving/day of dairy products had a dietary calcium level below 65% of the daily requirement. In contrast, individuals consuming ≥1 serving/day of dairy products met the recommendation for calcium. These results indicate that dairy products serve as a major source of people’s daily calcium intake in Korea. In addition, calcium from dairy products is absorbed more efficiently in the body compared with calcium from non-dairy products, because the phosphorylated serine and threonine residues in dairy products play a role in making calcium ion-soluble during the digestive processes [34]. These results may provide valid evidence for the public on the consumption of dairy products, which is the simplest and the most efficient way to achieve the daily calcium requirement. However, considering that nearly two thirds (59.0%) of Korean adults are not regularly consuming dairy products, further research needs to be conducted to explore differences in the major sources of dietary calcium between dairy product consumers and non-consumers, and a different strategy is needed for non-consumers to achieve their daily requirements for calcium.

In previous studies conducted in Western countries, it was reported that milk and dairy products are important components of a healthy diet. However, our findings showed that the consumption of milk and dairy products does not necessarily guarantee a healthy diet [1,35]. This coincides with the finding from studies on dietary patterns of Koreans reporting that milk and dairy products, which constitute a relatively marginal proportion in the Korean diet, are not distinctively characterized as a specific dietary pattern [36,37]. Additionally, dairy products are rarely consumed in the traditional Korean diet where the staple food is rice [38,39]. Milk and dairy products, predominantly, tend to be consumed together with bread rather than with rice. This is supported by previous studies on the dietary patterns of Koreans showing that dairy products and flour/breads follow the same dietary pattern [40,41].

We found that consuming more than the recommended amount of dairy products in a day reduced the risk of obesity and abdominal obesity. Results from this study, to an extent, confirmed findings from previous studies regarding the beneficial effects of dairy product consumption on MetS [12,25,42] and body weight/adiposity [43,44]. The large amount of dietary calcium obtained from dairy products reduces the concentrations of intracellular calcium regulators, parathyroid hormone and calcitriol [45]. The reduced level of intracellular calcium regulators is accompanied by a decreased transcription of the fatty acid synthase, which is essential for lipogenesis. This process inhibits lipogenesis while facilitating lipolysis, resulting in a breakdown of triglycerides in the adipose tissue [46]. Furthermore, a high consumption of dairy products and calcium leads to more fecal excretion of long-chain fatty acids (e.g., palmitic, stearic, and oleic acids) which leads to an increase in apoptosis [47]. The series of processes explained above decreases fat absorption in the body and reduces the risk of impaired glucose metabolism by preventing apoptosis of the pancreatic β-cells [47]. Although there is abundant literature on the associations of dairy consumption with risks of obesity, the findings of epidemiological studies are still confusing [48,49]. Further, preceding studies on dairy consumption in relation to obesity have found that this association was significantly modified by other factors such as calcium intake and weight status [50,51]. In other words, while inverse associations were observed between dairy products and obesity in individuals with a low intake of calcium or in obese individuals, these effects were not found in their respective counterparts. Additionally, it is not easy to identify the role of dairy product consumption in the development of obesity in Koreans who consume negligible amounts of milk and dairy products in their traditional diet, compared to those from Western countries who consume larger quantities of dairy products.

In this study, associations were found between consuming more than one serving of dairy products per day, and lower risks of elevated FBG and blood pressure. Evidence from observational and intervention studies demonstrated that dairy product consumption was helpful in maintaining an optimal level of blood glucose by increasing the levels of dairy protein-induced insulin [52,53]. In particular, branched-chain amino acids (e.g., leucine), which are abundant in dairy products, stimulate insulin secretions, resulting in effectively decreased postprandial glucose levels [54]. Additionally, dairy whey protein increases the levels of glucose-dependent insulinotropic peptide secreted by the endocrine K cells in the small intestine and also helps pancreatic β-cells with normal insulin secretion [55]. The findings from existing investigations regarding the anti-hypertension effects of dairy products are not unanimous [56,57]. However, in accordance with previous studies [58,59], we identified the role of dairy product consumption in lowering blood pressure in women, but not in men. Decreased blood pressure by dairy product consumption could be explained by the calcium-dependent and calcium-independent mechanisms. First, the calcium-dependent mechanism is connected to 1,25-dihydroxyvitamin D (calcitriol). Lowered serum calcium concentrations increase 1,25-dihydroxyvitamin D levels, resulting in a rapid inflow of calcium ions into the smooth cells, thus increasing the blood pressure [60]. However, the opposite might occur when enough calcium is supplied by dairy products to prevent increases in blood pressure. Another blood pressure-lowering mechanism is the calcium-independent mechanism, which is mediated by dairy proteins playing the role of angiotensin-converting enzyme (ACE) inhibitors [59]. There are two types of dairy proteins namely, casein and whey. These proteins could be hydrolyzed during the digestive processes into peptide fragments, casokinin and lactokinin, which function as ACE inhibitors in lowering blood pressure [61]. When blood pressure falls in the body, the rennin secreted from the kidney converts angiotensinogen to angiotensin I. Therefore, ACE converts inactive angiotensin I to active angiotensin II resulting in increased blood pressure through various pathways. In contrast, ACE inhibitors, when present, inhibit the conversion of inactive angiotensin I to active angiotensin II, because of which there is no increase in the blood pressure [62].

The beneficial effects of dairy products on health outcomes can be also partially explained by dairy lipids. The anti-inflammatory effects of dairy lipids such as polar lipids, conjugated linoleic acids, and polyunsaturated fatty acids were well-documented in a recent review by Lordan & Zabetakis [63]. In particular, dairy-derived polar lipids such as phospholipids and sphingolipids located in the milk fat globule membrane enhance anti-inflammatory activities, unlike polar lipids from other foods which increase various inflammatory markers [64]. Previous in vitro studies suggested that these milk-derived polar lipids may play important roles in inhibiting the platelet aggregation activated by the platelet-activating factor and worsening systematic inflammation [65]. Additionally, milk polar lipids can improve the function of intestinal barriers which plays a role in blocking the influx of endotoxins and reducing metabolic endotoxemia-induced inflammation [66].

However, the positive association between dairy product consumption and reduced risk of MetS was found only in women. Although previous studies have showed differences in the protective effect of dairy products against MetS, between the sexes, they were unable to provide a corresponding explanation for this difference [14,51,67]. However, several plausible explanations may be suggested. First, food group intakes making a significant contribution to an increase in the risk of MetS might differ by sex. Both sexes showed similar tendencies in food group intakes according to dairy product consumption, but there were distinct differences in the intake of certain food groups. In men, intakes of non-alcoholic beverages and sugars/sweets differed substantially according to the daily consumption of dairy products per day. This was largely consistent with the findings of previous studies on Koreans’ dietary patterns, where individuals with a lower intake of fats/sugars, instant noodles, and refined grains showed decreased risk of MetS [32,36]. A randomized controlled trial with adults having MetS conducted by Dugan et al. [68] demonstrated that the effect of a low-fat dairy diet on MetS differs depending on the sex. A 6-week low-fat dairy intervention improved the metabolic health of women alone. However, this study was unable to explain the differences in these associations between the sexes, since both sexes consumed the same types of dairy products. Only a quarter of the total participants in the present study consumed milk and dairy products. Moreover, most of the men and women who consumed more than one serving of dairy products per day predominantly consumed whole fat milk. However, reduced fat and low-fat milk accounted for 7–9% of their total consumption of dairy products, which is marginal. Thus, there was a limitation in investigating the relationship between reduced fat/low-fat milk and the risks of obesity and MetS, and it was also difficult to verify the difference in these associations between the two sexes. Second, one of the reasons may be the differences in the prevalence of obesity and MetS between the sexes. Investigation of the prevalence of MetS and its components according to the levels of dairy product consumption revealed significant differences between men and women; prevalence was mostly higher in men (data not shown). It is possible that such differences in the prevalence of obesity and MetS between men and women might have caused the difference in the effects of dairy consumption on the development of obesity and MetS. Third, another possible reason is that the serving size of dairy products leading to positive metabolic effects in the body may vary between the sexes. In other words, men may experience a protective effect of dairy product consumption on MetS development only when they consume more than one serving of dairy products per day because of the physical differences between men and women. Thus, future studies are warranted to clarify the dose-response relationships between dairy products and the risk of MetS in the Korean population. In accordance with these possibilities, the sex-specific associations of dairy products with MetS may be related to the associations between MetS and sociodemographic characteristics. A previous study reported a significant association between MetS and sociodemographics in women, but not in men [69,70]. Moreover, women with a high socio-economic status and education level are more likely to have healthier behavior and food choices compared with their counterparts [71,72], but the same is not true of men. Although men and women showed similar sociodemographic characteristics according to dairy product consumption, it is assumed that the sex-specific association of sociodemographic status with MetS may lead to a sex-related discrepancy in the association between dairy product consumption and MetS risk.

The strengths and limitations of this study should be noted. These findings can be generalized to Korean adults, since the study used a nationally representative sample of Korean adults. This is the most recent study to report dairy product consumption patterns in relation to food and nutrient intake, and the risks of obesity and MetS among the Korean adult population. Our results coincide with previous studies, indicating that a higher consumption of dairy products was associated with a lower risk of MetS. In addition, we confirmed that this association differs between both sexes. Although our findings support population-wide associations between dairy product consumption and MetS, it is important to note that the interpretation of causality between them is limited by the cross-sectional nature of our data. Moreover, the prevalence of dairy product consumption was quite low among our study participants, especially when compared to Western studies. Therefore, it is still not possible to draw firm conclusions regarding the protective effects of dairy product consumption on obesity and MetS in Korean adults. Finally, information obtained from 24-h dietary recalls was used in this study to overcome limitations of invalidated FFQs that were used in previous Korean studies. However, the 24-h dietary recall data also have limitations in reflecting the usual intake [73]. Recently, the KNHANES conducted a nutritional survey using a newly developed and validated FFQ, but these data have not yet been released. Therefore, further study will be required to investigate the associations of usual dairy product consumption with the risks of obesity and MetS using the new FFQ data after they are released to the public.

## 5. Conclusions

In summary, we found that very few Korean adults consume more than the recommended level of dairy products. An inverse association between dairy product consumption and MetS was observed for women, but not for men. We also found that this discrepancy in the association with MetS risk and the differences in the distribution of nutrients and food intakes could be influenced by the sex-specific association between sociodemographic characteristics and MetS. Our findings showed that consuming ≥1 serving/day of dairy products was significantly associated with reduced risks of obesity and MetS, and was sufficient to meet the daily calcium requirements. Therefore, public nutritional education should inform people regarding the potential favorable effects of dairy products and encourage them to consume higher quantities of dairy products. In addition, a nutrition policy should be framed to increase the frequency of dairy product consumption and the ratio of persons consuming dairy products, rather than merely elevating the recommended levels of dairy products. We believe there is a need for further investigation regarding the causal effect of dairy product consumption on weight gain and MetS development among the Korean population from a long-term perspective, with consideration for the differences between the sexes.

## Figures and Tables

**Table 1:** General characteristics of the study population by dairy product consumption in Korean adults, KNHANES 2010–2013 ^1^.

	Total	Dairy Product Consumption (Servings/Day)			*p* Value
		0	0< to <1	≥1	
	*n* (Wt’d % ^2^)	*n* (Wt’d %)	*n* (Wt’d %)	*n* (Wt’d %)	
**Total**	13,692 (100.00)	8079 (59.01)	2079 (15.18)	3534 (25.81)	
**Sex**					
**Men**	5375 (50.98)	3479 (54.47)	644 (42.61)	1252 (47.69)	<0.0001 **^,3^
**Women**	8317 (49.02)	4600 (45.53)	1435 (57.39)	2282 (52.31)	
**Age (year)**					
**20–29**	1863 (20.95)	913 (17.86)	314 (22.93)	636 (26.90)	<0.0001 **
**30–49**	6736 (50.61)	3814 (49.98)	1130 (53.63)	1792 (50.36)	
**50–64**	5093 (28.44)	3352 (32.16)	635 (23.44)	1106 (22.74)	
**Income**					
**Lowest**	3192 (25.58)	2045 (27.48)	431 (22.14)	716 (23.20)	0.0007 **
**Lower middle**	3503 (25.93)	2020 (25.11)	568 (27.38)	915 (26.99)	
**Upper middle**	3482 (24.88)	2052 (24.71)	524 (25.14)	906 (25.13)	
**Highest**	3515 (23.60)	1962 (22.70)	556 (25.33)	997 (24.68)	
**Education level**					
**≤Middle school**	3089 (18.67)	2183 (22.48)	345 (13.69)	561 (12.76)	<0.0001 **
**High school**	5336 (42.38)	3170 (42.68)	772 (40.25)	1394 (42.88)	
**≥College**	5267 (38.95)	2726 (34.84)	962 (46.06)	1579 (44.35)	
**Smoking**					
**No (non-/former smoker)**	10,860 (72.95)	6202 (69.70)	1722 (76.82)	2936 (78.21)	<0.0001 **
**Yes (current smoker)**	2832 (27.05)	1877 (30.30)	357 (23.18)	598 (21.79)	
**Alcohol consumption**					
**Never/rarely**	5925 (38.17)	3334 (36.21)	980 (41.26)	1611 (40.92)	<0.0001 **
**1–4/month**	4932 (38.12)	2834 (37.20)	751 (38.19)	1347 (40.19)	
**≥2/week**	2835 (23.71)	1911 (26.59)	348 (20.54)	576 (18.89)	
**Regular physical activity ^4^**					
**Yes**	6380 (48.39)	3674 (47.32)	920 (46.42)	1786 (51.96)	0.0001 **
**No**	7312 (51.61)	4405 (52.68)	1159 (53.58)	1748 (48.04)	
**Vitamin D status ^5^**					
**Deficient**	4044 (29.83)	2346 (29.22)	670 (31.81)	1028 (30.10)	0.0676
**Inadequate**	6547 (47.42)	3834 (47.21)	996 (48.12)	1717 (47.50)	
**Adequate**	3101 (22.75)	1899 (23.57)	413 (20.07)	789 (22.39)	

^1^ Data were from the Korea National Health and Nutrition Examination Surveys (KNHANES). All data except for sample size were weighted to account for the complex study design, according to the analytical guidelines of the KNHANES. ^2^ Wt’d %: Weighted %. ^3^ *p* value obtained from the Wald chi-square test for categorical variables (** *p* < 0.01). ^4^ Having physical activity was defined as walking ≥ 5 times a week for ≥30 min each time. ^5^ Vitamin D status was categorized as deficient (<12 ng/mL), inadequate (12–20 ng/mL), and adequate (>20 ng/mL) based on serum 25(OH)D levels [31].

**Table 2:** Energy and nutrient intakes by dairy product consumption in Korean adults, KNHANES 2010–2013 ^1^.

		Men (*n* = 5375)				Women (*n* = 8317)			
		Dairy Product Consumption (Servings/Day)			*p* Value	Dairy Product Consumption (Servings/Day)			*p* Value
	KDRI ^2^	0 (*n* = 3479)	0< to <1 (*n* = 644)	≥1 (*n* = 1252)		0 (*n* = 4600)	0< to <1 (*n* = 1435)	≥1 (*n* = 2282)	
		Mean ± SE	Mean ± SE	Mean ± SE		Mean ± SE	Mean ± SE	Mean ± SE	
**Total energy (kcal)**		2362.3 ± 19.0 ^3^	2352.8 ± 36.5	2539.2 ± 29.3	<0.0001 **^,4^	1738.4 ± 23.1	1799.2 ± 26.6	1919.9 ± 27.2	<0.0001 **
**Carbohydrate (g)**		357.77 ± 2.93	368.27 ± 5.98	381.73 ± 4.35	<0.0001 **	274.81 ± 3.54	281.02 ± 4.19	297.09 ± 4.58	<0.0001 **
**Carbohydrate (% of energy)**	55–70%	62.59 ± 0.29	63.48 ± 0.57	61.44 ± 0.40	0.0043 **	65.02 ± 0.45	63.87 ± 0.52	62.97 ± 0.48	<0.0001 **
**Protein (g)**		85.02 ± 0.96	82.95 ± 1.84	91.48 ± 1.33	<0.0001 **	62.73 ± 1.15	64.42 ± 1.28	70.02 ± 1.25	<0.0001 **
**Protein (% of energy)**	7–20%	14.27 ± 0.09	14.06 ± 0.19	14.36 ± 0.13	0.3828	14.30 ± 0.14	14.24 ± 0.17	14.50 ± 0.14	0.1212
**Fat (g)**		48.06 ± 0.72	49.10 ± 1.37	59.81 ± 1.17	<0.0001 **	37.08 ± 1.01	39.72 ± 1.11	45.34 ± 1.08	<0.0001 **
**Fat (% of energy)**	15–25%	17.60 ± 0.17	18.42 ± 0.35	20.53 ± 0.26	<0.0001 **	18.19 ± 0.29	19.22 ± 0.35	20.58 ± 0.32	<0.0001 **
**Dietary fiber (g) ^5^**	M: 25 g, W: 21 g	12.56 ±0.23	12.52 ± 0.43	12.35 ± 0.35	0.8570	10.85 ± 0.28	10.95 ± 0.34	10.47 ± 0.31	0.2271
**Sodium (mg) ^5^**	1100–1500 mg	5948.3 ± 66.9	5681.3 ± 121.6	5403.6 ± 90.8	<0.0001 **	4273.4 ± 76.6	4198.8 ± 112.3	3856.5 ± 88.7	<0.0001 **
**Potassium (mg) ^5^**		3493.0 ± 26.8	3496.7 ± 46.8	3628.7 ± 33.4	0.0022 **	2763.8 ± 32.5	2764.8 ± 39.0	2918.2 ± 35.9	<0.0001 **
**Phosphorous (mg) ^5^**		1346.8 ± 7.2	1365.9 ± 14.2	1481.8 ± 10.9	<0.0001 **	1000.2 ± 8.6	1028.9 ± 10.5	1139.2 ± 9.6	<0.0001 **
**Calcium (mg) ^5^**	700–750 mg	513.61 ± 6.05	556.61 ± 10.11	778.92 ± 10.18	<0.0001 **	393.84 ± 6.38	456.75 ± 8.47	649.8 ± 9.21	<0.0001 **
**Calcium from dairy products (mg) ^5^**		0.11 ± 0.85	73.48 ± 3.27	312.57 ± 6.88	<0.0001 **	1.51 ± 2.38	85.36 ± 3.55	307.29 ± 5.45	<0.0001 **
**Calcium from non-dairy products (mg) ^5^**		513.73 ± 5.97	483.06 ± 9.59	466.34 ± 7.80	<0.0001 **	392.33 ± 5.97	371.39 ± 7.82	342.52 ± 7.74	<0.0001 **

^1^ Data were from the Korea National Health and Nutrition Examination Surveys (KNHANES). All data were weighted to account for the complex study design, according to the analytical guidelines of the KNHANES. The multiple linear regression models included covariates including age (continuous), income (lowest, lowest middle, upper middle, and highest), education level (middle school graduates or less, high school graduates, and college graduation or higher), smoking status (non-/former smoker or current smoker), alcohol consumption (never/rarely, 1–4 times/month, and ≥2 times/week), and physical activity (yes or no). ^2^ KDRI, Dietary Reference Intakes for Koreans [9]. ^3^ All values represented means ± standard errors (SEs). ^4^ *p* value obtained from the linear regression analysis for continuous variables (** *p* < 0.01). ^5^ The models also included total daily energy (continuous) intake as an independent variable.

**Table 3:** Food group intakes according to dairy product consumption in Korean adults, KNHANES 2010–2013 ^1^.

	Men (*n* = 5375)					Women (*n* = 8317)		
	Dairy Product Consumption (Servings/Day)			*p* Value		Dairy Product Consumption (Servings/Day)		*p* Value
	0 (*n* = 3479)	0< to <1 (*n* = 644)	≥1 (*n* = 1252)		0 (*n* = 4600)	0< to <1 (*n* = 1435)	≥1 (*n* = 2282)	
	Mean ± SE	Mean ± SE	Mean ± SE		Mean ± SE	Mean ± SE	Mean ± SE	
**Whole grains (g/day)**	28.10 ± 1.36 ^2^	31.89 ± 2.72	28.56 ± 1.81	0.4256	23.50 ± 1.27	24.06 ± 1.88	25.34 ± 1.82	0.6292
**White rice and refined grains (g/day)**	239.65 ± 2.50	225.31 ± 5.21	210.76 ± 3.82	<0.0001 **^,3^	166.59 ± 2.81	151.17 ± 3.71	141.66 ± 3.44	<0.0001 **
**Noodles, dumplings, and instant ramen (g/day)**	46.69 ± 1.86	51.70 ± 3.96	46.00 ± 2.94	0.4411	39.75 ± 2.17	39.37 ± 2.98	33.92 ± 2.39	0.0502
**Flour, breads, cakes, and cookies (g/day)**	26.48 ± 1.40	35.61 ± 2.38	45.79 ± 2.41	<0.0001 **	25.35 ± 1.88	32.21 ± 2.32	33.21 ± 2.09	<0.0001 **
**Burgers, pizza, and sandwiches (g/day)**	4.47 ± 0.74	6.52 ± 2.19	5.13 ± 1.60	0.6686	5.45 ± 1.34	4.18 ± 1.52	5.20 ± 1.84	0.4898
**Starchy vegetables (g/day)**	34.76 ± 2.44	38.73 ± 4.00	36.73 ± 3.07	0.5771	34.73 ± 3.49	36.42 ± 3.59	37.49 ± 3.65	0.6676
**Other vegetables (g/day)**	410.75 ± 4.88	407.02 ± 9.79	368.77 ± 7.92	<0.0001 **	319.16 ± 5.71	329.12 ± 7.88	281.79 ± 6.73	<0.0001 **
**Fruits (g/day)**	161.32 ± 6.73	161.95 ± 9.90	154.70 ± 8.79	0.7873	177.85 ± 9.08	167.08 ± 9.68	176.30 ± 9.49	0.5074
**Meat/poultry (g/day)**	150.43 ± 5.66	126.60 ± 8.42	118.86 ± 6.40	0.0005 **	107.92 ± 7.03	95.44 ± 10.48	80.54 ± 7.26	<0.0001 **
**Fish and Shellfish (g/day)**	100.35 ± 3.12	86.28 ± 4.63	78.81 ± 3.77	<0.0001 **	72.47 ± 3.09	68.51 ± 3.79	61.08 ± 3.35	0.0006 **
**Eggs (g/day)**	30.31 ± 1.09	33.46 ± 2.26	30.98 ± 1.93	0.4257	21.83 ± 1.17	24.56 ± 1.77	23.24 ± 1.32	0.1887
**Legumes (g/day)**	34.79 ± 1.43	33.81 ± 3.13	32.98 ± 1.99	0.7325	24.16 ± 1.36	20.70 ± 1.77	23.11 ± 1.62	0.0942
**Nuts and seeds (g/day)**	6.88 ± 1.13	4.35 ± 0.67	4.52 ± 0.70	0.2107	4.46 ± 0.51	3.90 ± 0.65	5.33 ± 0.77	0.3755
**Sugars and sweets (g/day)**	11.69 ± 0.41	14.95 ± 1.03	13.18 ± 0.89	0.0058 **	8.70 ± 0.51	8.98 ± 0.71	8.30 ± 0.72	0.5768
**Oils and fats (g/day)**	11.16 ± 0.25	11.18 ± 0.54	9.77 ± 0.39	0.0052 **	7.81 ± 0.29	7.81 ± 0.38	6.54 ± 0.35	<0.0001 **
**Others (g/day)**	76.60 ± 1.54	70.11 ± 3.02	69.83 ± 2.49	0.0512	67.09 ± 10.87	63.50 ± 8.83	57.09 ± 8.54	0.0634
**Dairy products (g/day)**	0.58 ± 0.78	77.56 ± 2.77	317.67 ± 6.14	<0.0001 **	1.63 ± 2.23	81.89 ± 3.08	297.00 ± 4.64	<0.0001 **
**Whole-fat milk (g/day)**	−0.51 ± 0.82	32.38 ± 2.97	186.21 ± 7.27	<0.0001 **	1.54 ± 2.10	42.02 ± 3.07	168.30 ± 5.05	<0.0001 **
**Reduced fat (2%)/low-fat (1%) milk (g/day)**	−0.18 ± 0.51	3.65 ± 1.28	25.27 ± 3.23	<0.0001 **	−0.63 ± 0.97	6.12 ± 1.57	28.12 ± 2.23	<0.0001 **
**Sweetened milk (g/day)**	0.54 ± 0.42	1.49 ± 0.81	27.80 ± 2.99	<0.0001 **	−0.96 ± 0.96	−0.84 ± 1.04	15.21 ± 1.92	<0.0001 **
**Yogurt (g/day)**	0.03 ± 0.36	20.33 ± 1.91	49.65 ± 3.12	<0.0001 **	2.41 ± 1.96	20.15 ± 2.32	66.45 ± 4.13	<0.0001 **
**Cheese/cheese products (g/day)**	−0.03 ± 0.29	2.35 ± 0.44	1.64 ± 0.45	<0.0001 **	0.11 ± 0.15	2.26 ± 0.24	2.17 ± 0.28	<0.0001 **
**Ice cream/dairy-based desserts (g/day)**	0.44 ± 0.33	17.32 ± 1.68	26.91 ± 2.59	<0.0001 **	−1.00 ± 0.71	11.71 ± 1.22	16.70 ± 1.43	<0.0001 **
**Non-alcoholic beverages (g/day)**	151.75 ± 6.84	167.63 ± 13.72	130.58 ± 8.75	0.0346 *	124.01 ± 9.79	128.45 ± 11.87	126.17 ± 14.39	0.8505
**Alcoholic beverages (g/day)**	266.89 ± 9.71	191.07 ± 17.27	149.66 ± 12.70	<0.0001 **	154.80 ± 12.29	160.64 ± 13.88	126.27 ± 11.34	<0.0001 **

^1^ Data were from the Korea National Health and Nutrition Examination Surveys (KNHANES). All data were weighted to account for the complex study design according to the analytical guidelines of the KNHANES. The multiple linear regression models included covariates including age (continuous), income (lowest, lowest middle, upper middle, and highest), education level (middle school graduates or less, high school graduates, and college graduation or higher), smoking status (non-/former smoker or current smoker), alcohol consumption (never/rarely, 1–4 times/month, and ≥2 times/week), and physical activity (yes or no). ^2^ All values represented means ± standard errors (SEs) ^3^ *p* value obtained from the linear regression analysis for continuous variables (* *p* < 0.05, ** *p* < 0.01).

**Table 4:** Prevalence of obesity, metabolic syndrome, and its components according to dairy product consumption in Korean adults, KNHANES 2010–2013 ^1^.

	Total (*n* = 13,692)	Dairy Product Consumption (Servings/Day)			*p* Value
		0 (*n* = 8079)	0< to <1 (*n* = 2079)	≥1 (*n* = 3534)	
	*n* (Wt’d %)	*n* (Wt’d %)	*n* (Wt’d %)	*n* (Wt’d %)	
**Men**					
***n***	5375	3479	644	1252	
**Obesity ^2^**	2060 (37.90) ^3^	1361 (38.65)	219 (33.65)	480 (38.10)	0.1078
**MetS ^2^**	1426 (23.51)	982 (25.23)	152 (21.06)	292 (20.25)	0.0011 **^,4^
**Abdominal obesity ^2^**	1349 (24.13)	919 (25.38)	148 (21.60)	282 (22.11)	0.0487 *
**Lowered HDL-C ^2^**	1462 (25.64)	927 (25.68)	190 (26.81)	345 (24.96)	0.7144
**Elevated TG ^2^**	2287 (39.73)	1552 (41.79)	273 (40.85)	462 (33.79)	<0.0001 **
**Elevated FBG ^2^**	1793 (29.09)	1245 (31.53)	189 (24.17)	359 (25.18)	<0.0001 **
**Elevated BP ^2^**	1856 (30.78)	1283 (20.84)	195 (28.45)	378 (26.64)	0.0012 **
**Women**					
***N***	8317	4600	1435	2282	
**Obesity ^2^**	2194 (25.85)	1372 (28.89)	325 (23.17)	497 (21.45)	<0.0001 **
**MetS ^2^**	1792 (19.73)	1195 (23.76)	246 (16.02)	351 (13.98)	<0.0001 **
**Abdominal obesity ^2^**	2896 (33.23)	1816 (37.88)	431 (29.16)	649 (26.47)	<0.0001 **
**Lowered HDL-C ^2^**	3465 (40.54)	2017 (43.05)	589 (40.06)	859 (35.82)	<0.0001 **
**Elevated TG ^2^**	1978 (21.85)	1212 (24.29)	303 (19.42)	463 (18.50)	<0.0001 **
**Elevated FBG ^2^**	1579 (17.78)	1018 (20.78)	212 (13.77)	349 (14.29)	<0.0001 **
**Elevated BP ^2^**	1732 (17.98)	1115 (20.99)	247 (14.91)	370 (13.89)	<0.0001 **

^1^ Data were from the Korea National Health and Nutrition Examination Surveys (KNHANES). All data except for sample size were weighted to account for the complex study design according to the analytical guidelines of the KNHANES analytical guidelines. MetS, metabolic syndrome; HDL-C, HDL cholesterol; TG, triglyceride; FBG, fasting blood glucose; BP, blood pressure. ^2^ Definitions: obesity, BMI ≥ 25 kg/m^2^; MetS, having three or more of following risk factors: (1) abdominal obesity, waist circumference ≥ 90 cm (men) or ≥ 80 cm (women); (2) lowered HDL-C, fasting HDL-C < 40 mg/dL (men) or < 50 mg/dL (women); (3) elevated TG, fasting TG ≥ 150 mg/dL or specific treatment for hypertriglyceridemia; (4) elevated FBG, FBG ≥ 100 mg/dL or specific treatment for/previously diagnosed with type 2 diabetes mellitus; (5) elevated BP, systolic BP ≥ 130 mmHg or diastolic BP ≥ 85 mmHg or specific treatment for/previously diagnosed with hypertension. ^3^ Wt’d %: Weighted %. ^4^ *p* value obtained from the Wald chi-square test for categorical variables (* *p* < 0.05, ** *p* < 0.01).

**Table 5:** The adjusted odds ratios (AORs) and 95% confidence intervals (CIs) for obesity and MetS risk factors according to dairy product consumption in Korean adults, KNHANES 2010–2013 ^1^.

		Men (*n* = 5375)				Women (*n* = 8317)			
		Dairy Product Consumption (Servings/Day)			*p* for trend	Dairy Product Consumption (Servings/Day)			*p* for Trend
		0 (*n* = 3479)	0< to <1 (*n* = 644)	≥1 (*n* = 1252)		0 (*n* = 4600)	0< to <1 (*n* = 1435)	≥1 (*n* = 2282)	
			AOR (95% CI)	AOR (95% CI)			AOR (95% CI)	AOR (95% CI)	
**Obesity ^2^**	**Crude**	1.00	0.81 (0.66–0.99)	0.98 (0.83–1.15)	0.5377	1.00	0.76 (0.65–0.90)	0.66 (0.57–0.76)	<0.0001 **^,3^
	**Model 1**	1.00	0.79 (0.64–0.97)	0.99 (0.84–1.16)	0.5812	1.00	0.89 (0.75–1.06)	0.78 (0.67–0.90)	0.0014 **
	**Model 2**	1.00	0.79 (0.64–0.97)	0.98 (0.83–1.15)	0.5194	1.00	0.89 (0.75–1.06)	0.77 (0.66–0.89)	0.0010 **
**Abdominal obesity ^2^**	**Crude**	1.00	0.81 (0.65–1.01)	0.83 (0.69–1.00)	0.0337 *	1.00	0.68 (0.59–0.79)	0.58 (0.51–0.66)	<0.0001 **
	**Model 1**	1.00	0.83 (0.66–1.04)	0.83 (0.66–1.04)	0.1849	1.00	0.82 (0.70–0.97)	0.72 (0.62–0.82)	<0.0001 **
	**Model 2**	1.00	0.83 (0.66–1.04)	0.89 (0.74–1.08)	0.1539	1.00	0.82 (0.70–0.97)	0.71 (0.62–0.82)	<0.0001 **
**Lowered HDL-C ^2^**	**Crude**	1.00	1.06 (0.86–1.31)	0.96 (0.82–1.13)	0.7387	1.00	0.88 (0.76–1.02)	0.74 (0.65–0.84)	<0.0001 **
	**Model 1**	1.00	1.11 (0.89–1.39)	1.04 (0.88–1.23)	0.5786	1.00	0.95 (0.82–1.10)	0.80 (0.70–0.91)	0.0007 **
	**Model 2**	1.00	1.11 (0.89–1.39)	1.05 (0.89–1.24)	0.4936	1.00	0.95 (0.82–1.11)	0.82 (0.72–0.93)	0.0025 **
**Elevated TG ^2^**	**Crude**	1.00	0.97 (0.79–1.19)	0.71 (0.61–0.83)	<0.0001 **	1.00	0.75 (0.63–0.90)	0.71 (0.60–0.82)	<0.0001 **
	**Model 1**	1.00	1.08 (0.88–1.33)	0.86 (0.73–1.01)	0.1080	1.00	0.93 (0.78–1.14)	0.93 (0.80–1.09)	0.3599
	**Model 2**	1.00	1.08 (0.88–1.33)	0.86 (0.73–1.01)	0.1069	1.00	0.94 (0.77–1.14)	0.94 (0.80–1.10)	0.4118
**Elevated FBG ^2^**	**Crude**	1.00	0.70 (0.57–0.86)	0.73 (0.62–0.86)	<0.0001 **	1.00	0.61 (0.50–0.74)	0.64 (0.54–0.75)	<0.0001 **
	**Model 1**	1.00	0.84 (0.67–1.06)	0.98 (0.82–1.18)	0.6568	1.00	0.73 (0.60–0.89)	0.79 (0.67–0.93)	0.0021 **
	**Model 2**	1.00	0.84 (0.67–1.06)	0.98 (0.82–1.18)	0.6496	1.00	0.73 (0.60–0.90)	0.80 (0.67–0.95)	0.0030 **
**Elevated BP ^2^**	**Crude**	1.00	0.83 (0.66–1.04)	0.74 (0.62–0.87)	0.0004 **	1.00	0.65 (0.54–0.78)	0.61 (0.52–0.72)	<0.0001 **
	**Model 1**	1.00	0.99 (0.7801.26)	0.92 (0.77–1.10)	0.4066	1.00	0.86 (0.71–1.06)	0.85 (0.71–1.01)	0.2379
	**Model 2**	1.00	0.99 (0.78–1.26)	0.91 (0.76–1.10)	0.3869	1.00	0.86 (0.71–1.06)	0.85 (0.71–1.01)	0.2338
**MetS ^2^**	**Crude**	1.00	0.80 (0.64–1.01)	0.75 (0.63–0.89)	0.0005 **	1.00	0.62 (0.51–0.75)	0.51 (0.44–0.60)	<0.0001 **
	**Model 1**	1.00	0.93 (0.73–1.19)	0.95 (0.80–1.14)	0.5561	1.00	0.79 (0.64–0.98)	0.67 (0.56–0.80)	<0.0001 **
	**Model 2**	1.00	0.93 (0.73–1.19)	0.96 (0.80–1.14)	0.5829	1.00	0.79 (0.64–0.98)	0.67 (0.56–0.80)	<0.0001 **

^1^ Data were from the Korea National Health and Nutrition Examination Surveys (KNHANES). All data were weighted to account for the complex study design according to the analytical guidelines of the KNHANES. AOR, adjusted odds ratio; 95% CI, 95% confidence interval; HDL-C, HDL cholesterol; TG, triglyceride; FBG, fasting blood glucose; BP, blood pressure; MetS, metabolic syndrome. Multiple logistic regression analysis was performed to estimate the odds ratio for obesity, MetS, and its components for the study participants from the KNHANES 2010–2013, in three models: The crude model was unadjusted; Model 1 was adjusted for age (continuous), income (lowest, lowest middle, upper middle, and highest), education level (middle school graduates or less, high school graduates, and college graduation or higher), smoking status (non-/former smoker or current smoker), alcohol consumption (never/rarely, 1–4 times/month, and ≥2 times/week), and physical activity (yes or no); Model 2 was additionally adjusted for total energy intake (continuous). ^2^ Definitions: obesity, BMI ≥ 25 kg/m^2^; MetS, having three or more of following risk factors: (1) abdominal obesity, waist circumference ≥ 90 cm (men) or ≥ 80 cm (women); (2) lowered HDL-C, fasting HDL-C < 40 mg/dL (men) or < 50 mg/dL (women); (3) elevated TG, fasting TG ≥ 150 mg/dL or specific treatment for hypertriglyceridemia; (4) elevated FBG, FBG ≥ 100 mg/dL or specific treatment for/previously diagnosed with type 2 diabetes mellitus; (5) elevated BP, systolic BP ≥ 130 mmHg or diastolic BP ≥ 85 mmHg or specific treatment for/previously diagnosed with hypertension. ^3^ *p* value obtained from the multiple logistic regression model with diagnosis of obesity or MetS as the outcome variable (* *p* < 0.05, ** *p* < 0.01).
